# Supplementary material for: Influencing Factors on the Quality of Lymph Node Dissection for Stage IA Non-Small Cell Lung Cancer: A Retrospective Nationwide Cohort Study
Source: Cancers (Basel). 2024 Jan 13;16(2):346. doi: 10.3390/cancers16020346 (PMC10814584; doi:10.3390/cancers16020346)
Supplement: Supplementary file 1 [file cancers-16-00346-s001.zip › cancers-2801939-supplementary.pdf]

Supplementary Table S1. Patient baseline characteristics of full sample and propensity matched cohort.

	Full sample					Propensity matched cohort				
Patient Baseline Characteristics [a]	L-SMLND (n =1190)	Non-L-SMLND (n =3081)	Absolute difference	SDM	*p* value	L-SMLND (n =1184)	Non-L-SMLND (n =1184)	Absolute difference	SDM	*p* value
Age, median (IQR)	66 (IQR: 61 to 70)	66 (IQR: 61 to 70)	0	-0.003	0.755	66 (IQR: 61 to 70)	65 (IQR: 61 to 70)	1	0.03	0.219
Sex, *n* (%)					0.204					0.232
Male	568 (48)	1408 (46)	2	-0.77		566 (48)	537 (45)	3	0.05	
Female	622 (52)	1673 (54)	2	-0.96		618 (52)	647 (55)	3	-0.03	
CCI, median (IQR)	3 (IQR, 2 to 4)	3 (IQR, 2 to 4)	0	0.013	0.586	3 (IQR, 2 to 4)	3 (IQR, 2 to 4)	0	0.06	0.12
ThRCRI, *n* (%)				0	0.856				0.07	0.09
A	1012 (85)	2610 (84.7)	0.3			1007 (85.1)	1061 (89.6)	4.5		
B	175 (14.7)	469 (15.2)	0.5			174 (14.7)	123 (10.4)	4.3		
C	3 (0.3)	2 (0.1)	0.2			3 (0.3)	0	0.3		
COPD, *n* (%)	274 (23)	542 (18)	5	-0.62	<0.001 *	272 (23)	255 (22)	1	0.06	0.401
Surgeon's experience, *n* (%)				-0.73	0.004 *				0.004	0.934
Initial 50 lobectomies	660 (56)	1863 (61)	5			659 (56)	661 (56)	0		
Later lobectomies (>50)	530 (44)	1218 (39)	5			525 (44)	523 (44)	0		
Type of lobectomy, *n* (%)				0.2	<0.001 *				0.03	0.07

Right lower bilobectomy	13 (0.4)	1 (0.1)	0.3			0	0	0		
Right upper bilobectomy	9 (0.3)	5 (0.4)	0.1			0	0	0		
Left lower lobectomy	595 (19.3)	78 (6.6)	12.7			78 (6.6)	369 (31.2)	24.6		
Right lower lobectomy	485 (15.7)	254 (21.3)	5.6			254 (21.5)	164 (13.9)	7.6		
Left upper lobectomy	767 (24.9)	288 (24.2)	0.7			288 (24.3)	262 (22.1)	2.2		
Right upper lobectomy	946 (30.7)	469 (39.4)	8.7			469 (39.6)	317 (26.8)	12.8		
Middle lobectomy	266 (8.6)	95 (8)	0.6			95 (8)	72 (6.1)	1.9		

L-SMLND = lobe-specific mediastinal lymph node dissection; IQR = interquartile range; CCI = Charlson Comorbidity Index; ThRCRI = Thoracic Revised Cardiac Risk Index; COPD = Chronic obstructive pulmonary disease; SDM = Standardized mean difference. * Statistically significant ($p < 0.05$).
